# Cytokinin treatments affect the apical-basal patterning of the *Arabidopsis* gynoecium and resemble the effects of polar auxin transport inhibition

**DOI:** 10.3389/fpls.2014.00191

**Published:** 2014-05-14

**Authors:** Victor M. Zúñiga-Mayo, J. Irepan Reyes-Olalde, Nayelli Marsch-Martinez, Stefan de Folter

**Affiliations:** ^1^Laboratorio Nacional de Genómica para la Biodiversidad, Centro de Investigación y de Estudios Avanzados del Instituto Politécnico NacionalIrapuato, México; ^2^Departamento de Biotecnología y Bioquímica, Centro de Investigación y de Estudios Avanzados del Instituto Politécnico NacionalIrapuato, México

**Keywords:** apical-basal patterning, gynoecium, *Arabidopsis*, plant developmental biology, auxin, cytokinin

## Abstract

The apical-basal axis of the *Arabidopsis* gynoecium is established early during development and is divided into four elements from the bottom to the top: the gynophore, the ovary, the style, and the stigma. Currently, it is proposed that the hormone auxin plays a critical role in the correct apical-basal patterning through a concentration gradient from the apical to the basal part of the gynoecium, as chemical inhibition of polar auxin transport through 1-*N*-naphtylphtalamic acid (NPA) application, severely affects the apical-basal patterning of the gynoecium. In this work, we show that the apical-basal patterning of gynoecia is also sensitive to exogenous cytokinin (benzyl amino purine, BAP) application in a similar way as to NPA. BAP and NPA treatments were performed in different mutant backgrounds where either cytokinin perception or auxin transport and perception were affected. We observed that cytokinin and auxin signaling mutants are hypersensitive to NPA treatment, and auxin transport and signaling mutants are hypersensitive to BAP treatment. BAP effects in apical-basal gynoecium patterning are very similar to the effects of NPA, therefore, it is possible that BAP affects auxin transport in the gynoecium. Indeed, not only the cytokinin-response *TCS::GFP* marker, but also the auxin efflux carrier PIN1 (*PIN1::PIN1:GFP*) were both affected in BAP-induced valveless gynoecia, suggesting that the BAP treatment producing the morphological changes has an impact on both in the response pattern to cytokinin and on auxin transport. In summary, we show that cytokinin affects proper apical-basal gynoecium patterning in *Arabidopsis* in a similar way to the inhibition of polar auxin transport, and that auxin and cytokinin mutants and markers suggest a relation between both hormones in this process.

## INTRODUCTION

The gynoecium is the female reproductive organ of the flower. Different axes can be distinguished during the development of the *Arabidopsis thaliana* gynoecium and one of them is the apical-basal axis. This axis can be divided into four domains: the stigma at the apical part, consisting of a single layer of elongated cells called papillae, followed by a solid cylinder below, called the style, then there is the ovary which is the most complex part of the gynoecium and contains the ovules, and finally in the basal part the gynophore, which is a short stalk-like structure connecting the gynoecium with the rest of the plant (Balanza et al., 2006; Roeder and Yanofsky, 2006; Alvarez-Buylla et al., 2010).

Plants produce different hormones, which are involved in many developmental processes throughout their life cycle (Durbak et al., 2012; Lee et al., 2013). One of the most widely studied hormones is auxin (Tromas and Perrot-Rechenmann, 2010; Sauer et al., 2013). It has been reported that alterations in polar auxin transport, as occurs in the *pin1* mutant (Okada et al., 1991), or treatment with the polar auxin transport inhibitor 1-*N*-naphtylphtalamic acid (NPA; Nemhauser et al., 2000), or alterations in auxin signaling, occurring in the *ettin* mutant (Sessions and Zambryski, 1995), or deficiency in auxin biosynthesis, shown in the *yuc1 yuc4* (Cheng et al., 2006) and the *wei8 tar2* (Stepanova et al., 2008) mutants, have strong impact on gynoecium development, affecting the establishment of their apical-basal patterning. It has been proposed that auxins act through a gradient in the establishment of apical-basal patterning of the gynoecium, where the highest concentration of auxin is in the apical end and decreases towards the basal part of the gynoecium (Nemhauser et al., 2000), though modified views have evolved related to the presence of an auxin gradient (Ostergaard, 2009; Larsson et al., 2013). Alterations in the apical-basal patterning of the gynoecium are distinguished by an increase in the style and gynophore domain sizes at the expense of the ovary, which in severe cases even completely disappears.

Another well-studied plant hormone is cytokinin, which is involved in different developmental processes such as shoot meristem formation and maintenance, organ formation, and seed germination, among others (Mok and Mok, 2001; Hwang et al., 2012; El-Showk et al., 2013). Recently, it has been reported that cytokinins are involved in the regulation of floral organ size, ovule number, and ovule development in the gynoecium (Bartrina et al., 2011; Bencivenga et al., 2012). Furthermore, cytokinins are involved in medial tissue proliferation at early stages of the developing gynoecium and at more mature stages in valve margin differentiation (Marsch-Martinez et al., 2012a, b; Reyes-Olalde et al., 2013).

In recent years special attention has been paid to the study of interactions between different hormones. Hormonal crosstalk provides an extra level of regulation in biological processes conferring robustness and stability, as well as flexibility (Moubayidin et al., 2009; Wolters and Jurgens, 2009; Depuydt and Hardtke, 2011; Vanstraelen and Benkova, 2012). The cytokinin–auxin crosstalk is important for the establishment and maintenance of the root apical meristem (RAM) and the shoot apical meristem (SAM). These two hormones act antagonistically in the RAM, cytokinin by promoting cell differentiation and auxin by promoting cell division (Dello Ioio et al., 2007; Ruzicka et al., 2009). Conversely, in the SAM, auxin increases cytokinin response through the repression of cytokinin signaling repressors (Zhao et al., 2010). Several studies have demonstrated that the cytokinin–auxin crosstalk can occur at different levels, cytokinin can affect auxin synthesis, transport or signaling, and *vice versa*, auxin can affect cytokinin synthesis, degradation, or signaling (Hwang et al., 2012; El-Showk et al., 2013).

Despite the large number of studies on the role of cytokinins in plant development, their functions in gynoecium development are just beginning to be explored (Marsch-Martinez et al., 2012a; Reyes-Olalde et al., 2013), while it’s possible interactions with other hormones in this organ have not been studied yet. In this study we analyzed the possible role of cytokinin in apical-basal patterning of the gynoecium and its possible interaction with auxin through exogenous application of the cytokinin benzyl amino purine (BAP) and the auxin transport inhibitor NPA to different mutants and cytokinin and auxin signaling markers. The results suggest that cytokinins are also involved in apical-basal patterning of the gynoecium, which is more evident when the auxin transport or signaling is affected.

## MATERIALS AND METHODS

### PLANT GROWTH CONDITIONS

All wild type and mutant plants used in this study are *Arabidopsis thaliana* ecotype Columbia. Plants were germinated in soil under long-day conditions (16–8 h, light–dark) in a growth chamber at 22°C. One week after germination, the plants were transferred to the greenhouse with a temperature range from 22 to 28°C, long-day conditions (13–11 h, light–dark approximately) and natural light.

### HORMONE TREATMENTS

One week after bolting, wild type, mutant and marker line inflorescences were dipped five consecutive days in BAP, NPA, or mock solutions. The BAP and NPA solutions contained 100 μM benzylaminopurine (BAP; Duchefa Biochemie, http://www.duchefa.com) or 100 μM NPA (Sigma–Aldrich, St. Louis, MO, USA) respectively, and 0.01% Silwet L-77 (Lehle Seeds, Round Rock, TX, USA). The mock solution contained only 0.01% Silwet L-77. All treated plants with their respective controls were grown simultaneously under the same conditions. For each mutant background five plants were treated, of which 10–15 main and secondary inflorescences were analyzed. The gynoecia were analyzed after anthesis. The treated plants were frequently monitored; the apical-basal patterning phenotypes began to be observed after 2 weeks.

The standard deviation was calculated considering the phenotype frequency percentages between each inflorescence analyzed. To determine whether there was a significant difference in the different phenotypes between wild type plants and the different treated mutants a Student’s *t*-test was performed comparing the phenotype frequency percentages of each mutant background versus wild type plants. The treatments for each mutant were performed twice with similar results. The results presented here are from one experiment.

### MICROSCOPY

For light pictures and phenotype analysis the plant material was dissected and observed using a Leica EZ4 D stereomicroscope (Leica, Wetzlar, Germany). Scanning electron microscopy images were captured using a Zeiss EVO40 environmental scanning electron microscope (Carl Zeiss, Oberkochen, Germany) with a 20 kV beam, and the signal was collected using the BSD detector, for which plant tissue was collected and directly observed in the microscope. For fluorescent microscopy, the images were captured using a LSM 510 META confocal scanning laser inverted microscope (Carl Zeiss, Oberkochen, Germany). Propidium iodide (PI) was excited using a 514-nm line and GFP was excited using a 488-nm line of an Argon laser. PI emission was filtered with a 575-nm longpass (LP) filter and GFP emission was filtered with a 500–550-nm bandpass (BP) filter.

## RESULTS

### EXOGENOUS APPLICATION OF CYTOKININ AFFECTS THE APICAL-BASAL PATTERNING OF THE *Arabidopsis* GYNOECIUM

Recently, we reported that cytokinins are important for the proliferation at the medial tissues in the gynoecium and for proper valve margin differentiation in *Arabidopsis* fruits (Marsch-Martinez et al., 2012a). It has been shown that auxin plays an important role in establishing the correct apical-basal patterning of the gynoecium (Nemhauser et al., 2000). Furthermore, it is known that cytokinin and auxin cross-talk at different levels in several developmental processes (El-Showk et al., 2013). With this in mind, we decided to analyze the effect of exogenous cytokinin applications on the apical-basal patterning of the *Arabidopsis* gynoecium. Inflorescences of wild type plants were treated once a day for a period of 5 days with 100 μM BAP solution. In parallel, we carried out a treatment with 100 μM NPA under the same conditions; this compound blocks the polar auxin transport, causing apical-basal patterning defects in the gynoecium (Nemhauser et al., 2000). This treatment was performed in order to compare the effect of exogenous cytokinin application versus polar auxin transport blocking.

We previously reported that prolonged BAP application (3–4 weeks) produced gynoecia with conspicuous tissue proliferation (Marsch-Martinez et al., 2012a). However, when the wild type inflorescences were treated with BAP during a shorter time (5 days) a gradient of phenotypes were observed. The first open flowers (flowers 1–5) after the treatment contained gynoecia with no obvious phenotype. The next floral buds to open (flowers 6–18) contained gynoecia that showed the proliferation that was reported previously. However, floral buds that opened later (flowers 19–31) contained gynoecia that showed apical-basal defects which are the focus of this study. In some cases we observed gynoecia with both phenotypes, the proliferation and the apical-basal defects; these gynoecia were developed in the transition zone of these two phenotypes. Finally normal gynoecia were developed.

**FIGURE 1:**
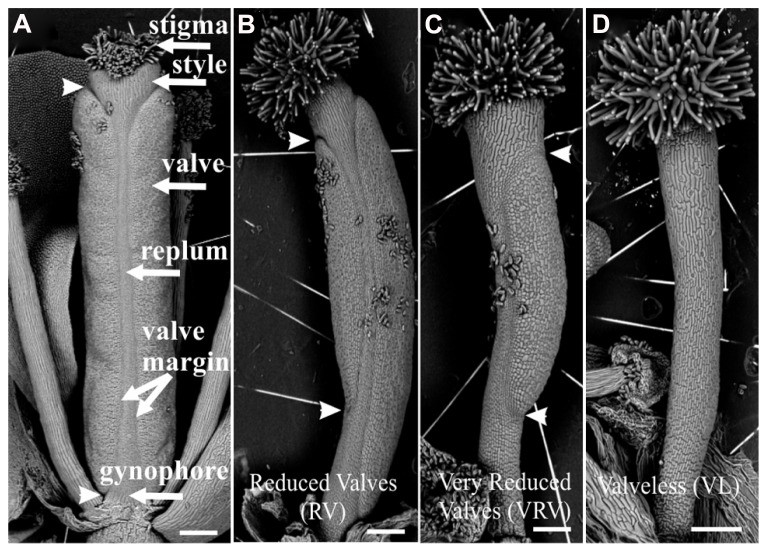
**Scanning electron micrographs of classification of apical-basal phenotypes in the *Arabidopsis* gynoecium. (A)** Mock-treated wild type gynoecium. **(B)** Gynoecium presenting a “Reduced Valves” (RV) phenotype. **(C)** Gynoecium with the “Very Reduced Valves” (VRV) phenotype. **(D)** Gynoecium with the “Valveless” (VL) phenotype. These gynoecia were treated with NPA. The arrowheads indicate the beginning and the end of valves. Scale bars: **(A–D)** 200 μm.

**FIGURE 2:**
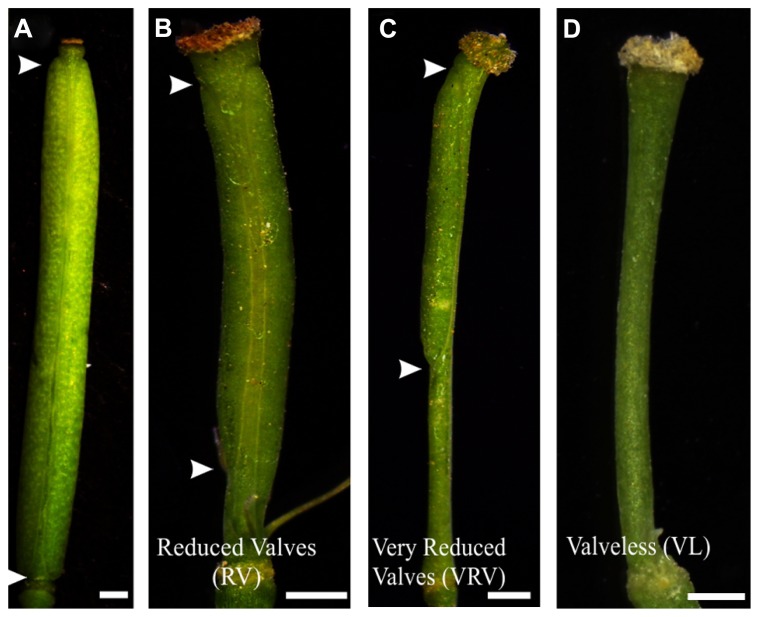
**Apical-basal phenotypes caused by exogenous BAP application. (A)** Mock-treated wild type gynoecium. **(B)** A gynoecium with the “Reduced Valves” (RV) phenotype. **(C)** Gynoecium with a “Very Reduced Valves” (VRV) phenotype. **(D)** Gynoecium with the “Valveless” (VL) phenotype. The arrowheads indicate the beginning and the end of valves. Scale bars: **(A)** 1 mm; **(B,C)** 400 μm; **(D)** 200 μm.

Two weeks after each treatment, the gynoecia of treated floral buds were analyzed. In both cases for wild type plants twelve to fifteen gynoecia per inflorescence showed apical-basal defects with different severities. The observed phenotypes were classified according to previously reported by Sohlberg et al. (2006). The classification consists of three categories based on valve development: (1) If the length of the valves was more than 50% the length of the gynoecium, but less than the length of valves of mock-treated gynoecium, were named “reduced valves”; (2) This category includes gynoecia with one valve and gynoecia with two small valves that occupied less than half of its length; and (3) If the gynoecium did not develop any valves the phenotype was named “valveless” (**Figures 1 and 2**). 

**FIGURE 3:**
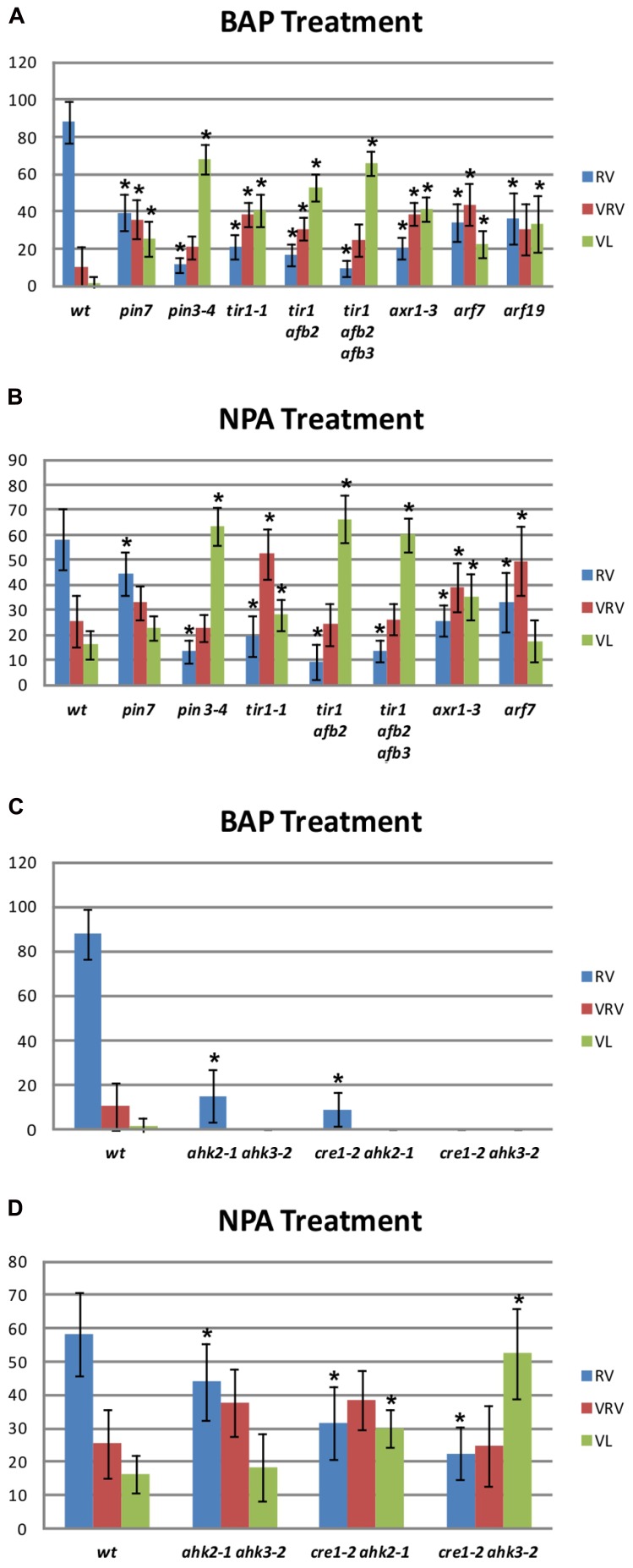
**Apical-basal gynoecium patterning phenotype frequency of NPA and BAP treatments in wild type and mutant backgrounds. (A,C)** Distribution of the different categories of apical-basal phenotypes in BAP-treated gynoecia. **(A)** Auxin signaling mutants. Col, *n* = 204; *pin7*, *n* = 111; *pin3*, *n* = 204; *tir1*, *n* = 145; *tir1 afb2*, *n* = 299; *tir1 afb2 afb3*, *n* = 383; *axr1*, *n* = 372; *arf7*, *n* = 122. **(C)** Cytokinin signaling mutants. *ahk2 ahk3*, *n* = 224; *cre1 ahk2*, *n* = 288; *cre1 ahk3*, *n* = 495. **(B,D)** Distribution of the different categories of apical-basal phenotypes in NPA-treated gynoecia. **(B)** Auxin signaling mutants Col, *n* = 231; *pin7*, *n* = 225; *pin3*, *n* = 258; *tir1*, *n* = 314; *tir1 afb2*, *n* = 557; *tir1 afb2 afb3*, *n* = 889; *axr1*, *n* = 406; *arf7*, *n* = 317; *arf19*, *n* = 434. **(C)** Cytokinin signaling mutants *ahk2 ahk3*, *n* = 163; *cre1 ahk2*, *n* = 148; *cre1 ahk3*, *n* = 177. RV, Reduced Valves; VRV, Very Reduced Valves; VL, Valveless. Error bars represent standard deviation. The “*n*” indicates the total number of analyzed gynoecia for each background. Values on the *y*-axis are percentages. The asterisk (*) indicates significant difference.

The BAP-treated wild type gynoecia presenting apical-basal defects were analyzed, and the majority of them (88%) showed reduced valves, 10% developed very reduced valves and almost 2% were classified as valveless (**Figures 2 and 3A**). In the case of NPA-treated wild type gynoecia, 59% of them showed reduced valves, 25% developed very reduced valves, and 16% showed the valveless phenotype (**Figure 3B**). The data obtained for the NPA treatment (**Figures 1 and 3**) are similar to those previously reported (Sohlberg et al., 2006). Comparing the frequencies of the phenotypes in both treatments, the defects observed due to BAP are less severe than the defects due to NPA, however, the occurrence of these phenotypes are constant between BAP treatments and significantly higher than the frequency in which they appear in untreated plants. These results indicate that, like NPA, exogenously applied cytokinin affects proper establishment of the apical-basal patterning in the *Arabidopsis* gynoecium.

### BAP AND NPA APPLICATIONS HAVE SIMILAR EFFECTS IN AUXIN TRANSPORT AND SIGNALING MUTANTS

It has been reported that the apical-basal gynoecium patterning of auxin biosynthesis or signaling mutants gynoecia is hypersensitive to NPA treatment (Staldal et al., 2008). In order to know whether the the BAP effect on the apical-basal patterning was related with any auxin related processes, we performed BAP treatments in different auxin transport and signaling mutants.

In *Arabidopsis*, polar auxin transport requires the activity of polarly localized PIN-FORMED (PIN) auxin efflux transporters (Benkova et al., 2003; Friml, 2003). The *pin1* mutant produces hardly any flowers (Okada et al., 1991), so it was discarded for this study. On the other hand, the *pin3 pin7* double mutant gynoecia show alterations in apical-basal patterning, but its reproductive development is also severely affected (Benkova et al., 2003). However, the *pin3* and *pin7* single mutants do not exhibit visible apical-basal defects. Therefore, these two mutants represent an opportunity to explore the effect of BAP application in a background where polar auxin transport is affected but development is not severely altered. When the *pin7* mutant was treated with BAP, 39% of gynoecia developed reduced valves, 37% developed very reduced valves, and 24% showed the valveless phenotype (**Figure 3A**). In the *pin3* mutant 11% of gynoecia showed reduced valves, 21% developed very reduced valves, and 68% showed the valveless phenotype (**Figure 3A**). These same mutants were also treated with NPA (**Figure 3B**). In the *pin7* mutant 22% of gynoecia did not develop valves, whereas this alteration was observed in 64% of *pin3* mutant gynoecia. These results indicate that the apical-basal patterning of *pin3* and *pin7* gynoecia is hypersensitive to both treatments and the valveless phenotype frequencies are similar for both treatments in the same mutant. In addition, the *pin3* mutant appears to be more sensitive than the *pin7* mutant to both treatments, suggesting that PIN3 plays a more relevant role in the establishment of apical-basal gynoecium patterning than PIN7.Furthermore, auxin signaling mutants were treated with BAP or NPA. First, different auxin receptor mutants were treated: the single mutant *transport inhibitor response 1* (*tir1*; Ruegger et al., 1998), the double mutant *tir1 auxin signaling F-box protein 2* (*afb2*), and the triple mutant *tir1 afb2 afb3* (Dharmasiri et al., 2005). The untreated *tir1* and *tir1 afb2* gynoecia did not exhibit obvious apical-basal defects, while *tir1 afb2 afb3* gynoecia occasionally showed apical-basal defects under our growth conditions. However, all three genotypes were hypersensitive to BAP treatment, and the frequency of the more severe phenotype (valveless) increased when auxin perception decreased, such that in *tir1, tir1 afb2*, and *tir1 afb2 afb3* plants 40, 53, and 64% of gynoecia, respectively, showed the valveless phenotype (**Figure 3A**). When the mutants were treated with NPA, in *tir1, tir1 afb2*, and *tir1 afb2 afb3* plants 28, 66, and 61% of gynoecia, respectively, showed the valveless phenotype (**Figure 3B**), indicating that these mutants are also hypersensitive to the NPA treatment.

In addition, mutants affected in auxin signaling, downstream perception, were treated with BAP and NPA. These mutants were *auxin resistant 1* (*axr1*), where a protein related to the ubiquitin-activating enzyme E1 is affected, and *auxin response factor 7* (*arf7*) and *arf19* mutants, where transcription factors that mediate auxin response are affected (Leyser et al., 1993; Harper et al., 2000; Okushima et al., 2005). Untreated *axr1* gynoecia occasionally showed apical-basal defects under our growth conditions, but this was not observed for *arf7* and *arf19*. Regarding the BAP treatment, the *axr1* mutant developed 41%, the *arf7* mutant 24%, and the *arf19* mutant 35% of gynoecia without valves (**Figure 3A**). These results indicate that these three mutants are hypersensitive to the BAP treatment. In the case of the NPA treatment, the *axr1* mutant developed 34% and the *arf7* mutant 18% of valveless gynoecia (**Figure 3B**), indicating that *axr1* is hypersensitive to NPA treatment. For the *arf19* mutant no data were obtained due to technical reasons.

In summary, the results indicate that the gynoecia of auxin transport and signaling mutants are hypersensitive to BAP application, resulting in apical-basal patterning defects. This phenomenon was already reported for NPA application (Staldal et al., 2008), therefore in this study NPA was used as reference, and produced similar results as seen for the BAP application.

### THE ABSENCE OF CYTOKININ RECEPTORS ALTERS THE RESPONSE TO BAP AND NPA APPLICATIONS

The above results suggest that disruption of auxin transport or signaling has an impact on the effect caused by BAP treatments on the apical-basal patterning of the gynoecium, as had been reported and was also observed here for NPA treatments. The next step was to explore the possibility that disturbances in processes related to cytokinin perception might also have an impact on the effect of these treatments. For this purpose, the *cytokinin response 1* (*cre1*) *Arabidopsis histidine kinase 2* (*ahk2*), *cre1 ahk3*, and *ahk2 ahk3* cytokinin receptor double mutants (Higuchi et al., 2004; Nishimura et al., 2004) were treated. Untreated double mutant gynoecia never presented apical-basal defects under our growth conditions. After BAP treatment, two of the three cytokinin receptor double mutants showed slight apical-basal defects, but none of them developed gynoecia with severe apical-basal phenotypes. In *ahk2 ahk3* and *cre1 ahk2* mutants 15 and 9% of gynoecia developed reduced valves, respectively (**Figure 3C**). The *cre1 ahk3* mutant gynoecia did not show visible apical-basal phenotypes (**Figure 3C**). These results suggest that the cytokinin receptors CRE1, AHK2, and AHK3 are required for the full effect of exogenous BAP application on the establishment of apical-basal patterning of gynoecia observed in wild type plants. An opposite response was observed when the cytokinin receptor mutants were treated with NPA. In the *ahk2 ahk3*, *cre1 ahk2*, and *cre1 ahk3* mutants 19, 30, and 53% of the gynoecia, respectively, showed the severe valveless phenotype (**Figure 3D**), in comparison to only 16% in wild type plants. These results suggest that adequate cytokinin perception is necessary to attenuate the impact of the reduction in polar auxin transport on the establishment of apical-basal patterning of the gynoecium.

### BAP AND NPA APPLICATIONS AFFECT THE EXPRESSION PATTERN OF CYTOKININ (*TCS::GFP*) AND AUXIN-RESPONSE MARKERS (*DR5::GFP*) AND THE AUXIN TRANSPORTER PIN1 (*PIN1::PIN1:GFP*) IN THE GYNOECIUM

It has been described that the cytokinin (*TCS::GFP*) and auxin-response (*DR5::GFP*) markers have well defined and mutually exclusive expression patterns in some regions of the gynoecium during development (Marsch-Martinez et al., 2012a). Besides, the auxin efflux carrier PIN1 is important for gynoecium development, because the *pin1* mutant produces almost no flowers and when flowers are produced their gynoecium show severe apical-basal patterning defects (Okada et al., 1991). We analyzed whether BAP or NPA application were able to cause changes in the expression pattern of PIN1 and the hormonal-response markers, and whether these changes could be related to the apical-basal gynoecium defects due to these treatments. For this purpose, each marker line was treated once a day for a period of 5 days, as done for the treatments described above, with the BAP or NPA solution for *TCS::GFP* and *DR5::GFP* and with BAP for *PIN1::PIN1:GFP*. The expression patterns of these marker lines were analyzed using confocal laser scanning microscopy when gynoecia with apical-basal defects were observed.

**FIGURE 4:**
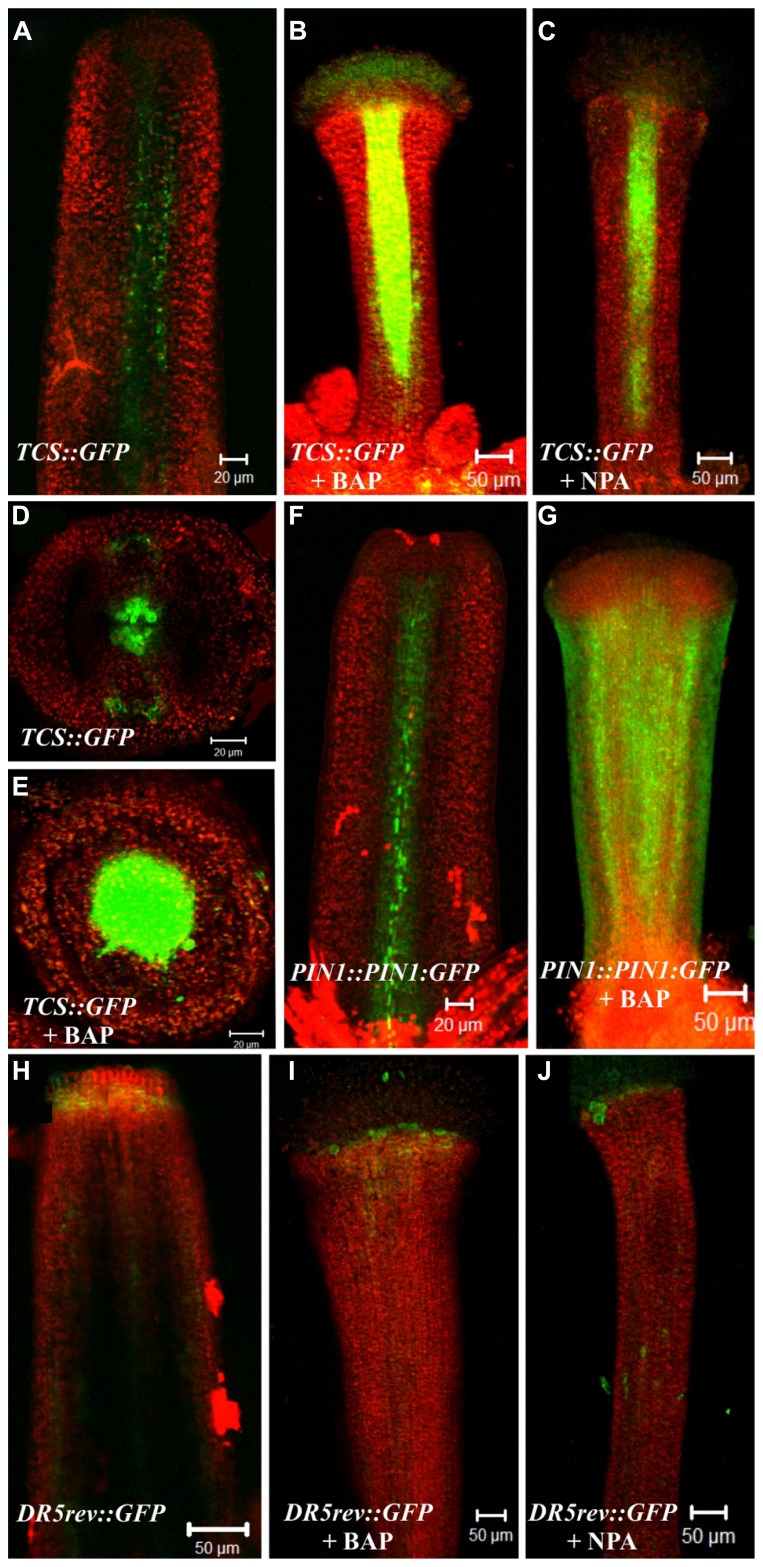
**Effect of cytokinin (BAP) and NPA application on the PIN1 (*PIN1::PIN1:GFP*), cytokinin (*TCS::GFP*) and the auxin-response markers (*DR5::GFP*). (A–E)** The fluorescence signal of the cytokinin response marker *TCS::GFP* observed in the wild type gynoecium at floral stage 10 in a longitudinal view **(A)** and transverse view **(D)**. Valveless gynoecium at floral stage 11 caused by BAP treatment in a longitudinal view **(B)** and transverse view **(E)**. Valveless gynoecium at floral stage 11 caused by NPA treatment in a longitudinal view **(C)**. **(F,G)** The fluorescence signal detection of the PIN1 marker *PIN1::PIN1:GFP* observed in the wild type gynoecium at floral stage 10 **(F)**. Valveless gynoecium at floral stage 10 caused by BAP treatment **(G)**. **(H–J)** The fluorescence signal detection of the auxin response marker *DR5::GFP* observed in wild type gynoecium at stage 12 **(H)**. Valveless gynoecium at floral stage 12 caused by BAP treatment **(I)**. Valveless gynoecium at floral stage 12 caused by NPA treatment **(J)**. Scale bars: **(A,D–F)** 20 μm; **(B,C,G–J)** 50 μm.

In wild type gynoecia between floral stages 8–10 (Smyth et al., 1990) the TCS::GFP signal was observed at the center, where the medial tissues are developing from the carpel marginal meristem (CMM), as we have observed before (Marsch-Martinez et al., 2012a; **Figures 4A,D**). After BAP or NPA treatment, the TCS::GFP signal was increased in the central zone of valveless gynoecia. However, these gynoecia had reduced development of the internal medial tissues (**Figures 4B,C,E**). For the DR5::GFP auxin-response marker in untreated gynoecia between stages 9–12 the signal was observed at the apical end of gynoecia and in the vasculature, as we have observed before (Marsch-Martinez et al., 2012a; **Figure 4H**). After BAP or NPA treatment, the DR5::GFP signal did not show obvious changes in these experiments (**Figures 4I,J**). However, in the wild type gynoecium at stage 10, the auxin efflux carrier PIN1 is expressed in the tissue that will give rise to the replum (**Figure 4F**), and after BAP treatment the *PIN1::PIN1:GFP* signal was observed in the whole valveless gynoecium (**Figure 4G**).

In summary, BAP and NPA application had comparable effects in the hormone reporter lines, this is, an increase in TCS::GFP activity in the central region of the gynoecium, but no detectable change in the DR5::GFP signal. Moreover, BAP application caused an increase in expression level and alteration of the localization of PIN1 in the gynoecium. These results correlate well with the observation that BAP and NPA treatments cause similar apical-basal patterning defects.

## DISCUSSION

### IMPACT OF CYTOKININ AND NPA APPLICATION ON APICAL-BASAL GYNOECIUM PATTERNING IN AUXIN TRANSPORT AND SIGNALING MUTANTS

Cytokinin is involved in different developmental processes throughout the *Arabidopsis* life cycle (Hwang et al., 2012; El-Showk et al., 2013), including proper gynoecium and fruit development (Marsch-Martinez et al., 2012a, b; Reyes-Olalde et al., 2013). Here, we evaluated the effect of exogenous cytokinin application on the establishment of apical-basal patterning of the *Arabidopsis* gynoecium.

BAP-treated gynoecia present the same apical-basal defects observed as when treated with NPA, but the frequencies in which altered phenotypes are observed are lower. Because the role of NPA is to block polar auxin transport and the phenotypes caused by both BAP and NPA treatments are similar, the results suggest that exogenously applied cytokinin might affect polar auxin transport and thereby cause the observed patterning phenotypes.

It has been reported that auxin biosynthesis or signaling mutant gynoecia are hypersensitive to NPA treatment in regard to apical-basal patterning (Staldal et al., 2008). In this study, we observed that the auxin transport mutants *pin3* and *pin7* were hypersensitive to both BAP and NPA treatments, and the sensitivity level was similar between treatments but different between mutants. In this case, the *pin3* mutant was more sensitive to either treatment compared to the *pin7* mutant, indicating that in the absence of the PIN3 function the imbalance caused by both BAP and NPA application has a greater impact on the establishment of apical-basal gynoecium patterning. This suggests that PIN3 and PIN7 contribute to different extent to proper gynoecium apical-basal patterning.

Furthermore, the different auxin signaling mutants analyzed in this study were also sensitive to both treatments. In the case of the auxin receptor mutants, only the mock-treated *tir1 afb2 afb3* gynoecia occasionally showed some apical-basal gynoecium patterning defects. However, the three different mutants were hypersensitive to BAP and NPA, suggesting that the proper establishment of the apical-basal gynoecium pattern is a robust process that even when auxin perception is severely affected can be carried out without major defects. However, when perturbations such as those caused by cytokinin application or by auxin transport inhibition occur, it becomes evident that a change in the level of auxin perception affects proper gynoecium development.

Auxin Response Factors (ARFs) are transcription factors that regulate transcription in an auxin-dependent manner. It is known that the *ARF7* and *ARF19* genes are involved in cell growth of leaves and in lateral root formation (Wilmoth et al., 2005; Okushima et al., 2007), and *ARF7* acts redundantly with *MONOPTEROS* (*MP/ARF5*) in the axial patterning of the embryo (Hardtke et al., 2004). We observed that the *arf7* and *arf19* mutants are hypersensitive to BAP application regarding apical-basal gynoecium patterning, suggesting a role of these genes in this process.

### IMPACT OF CYTOKININ AND NPA APPLICATION ON APICAL-BASAL GYNOECIUM PATTERNING IN CYTOKININ SIGNALING MUTANTS

When the cytokinin receptor mutants were treated with BAP, less severe or no alterations were observed in apical-basal gynoecium patterning, suggesting that the exogenous cytokinin needs to be perceived by the plant to trigger these changes. Interestingly, the altered apical-basal patterning phenotypes caused by NPA treatments were increased in the cytokinin receptor mutants.

A comparison of the effects of both treatments in the different cytokinin receptor mutant backgrounds, suggested a negative correlation between the ability to respond to cytokinin and the severity of the phenotype caused by auxin transport inhibition. In the mutants where cytokinin perception was more affected, i.e., less alteration in patterning caused by BAP (least phenotypic effect observed in *cre1 ahk3*), the effect of NPA was increased, i.e., more visible alterations in patterning.

This may indicate that cytokinin (perception) buffers the effect of decreased auxin polar transport in apical-basal patterning.

### IMPACT OF CYTOKININ AND NPA APPLICATION ON CYTOKININ (*TCS::GFP*) AND AUXIN-RESPONSE MARKERS (*DR5::GFP*) AND THE AUXIN TRANSPORTER PIN1 (*PIN1::PIN1:GFP*) IN THE GYNOECIUM

The cytokinin (*TCS::GFP*) and auxin-response (*DR5::GFP*) and PIN1 (*PIN1::PIN1:GFP*), markers were analyzed in gynoecia presenting apical-basal defects. The TCS::GFP signal was detected in the medial tissues during normal gynoecium development at early stages. We followed the TCS::GFP signal in the BAP and NPA induced valveless gynoecia. In these gynoecia the medial tissue showed reduced development. However, the TCS::GFP signal was not only maintained, but interestingly, it was increased.

NPA treatments have been shown to inhibit the formation of lateral organs in shoot apical meristems (Reinhardt et al., 2000). The valves of gynoecia are considered lateral organs (Benkova et al., 2003), and NPA has a comparable effect, producing valveless gynoecia. In the shoot apical meristem context, NPA does not affect the meristematic activity as shown by the maintenance of the activity of various meristem markers (Reinhardt et al., 2000). At the gynoecium, the activity of the *TCS::GFP* marker suggests that a similar situation occurs in this tissue, i.e., that the valves are not formed, but the meristematic activity at the medial tissues continues. Interestingly, the cytokinin signaling was not only maintained after the NPA treatment, but seemed to increase, as revealed by the increased fluorescence observed at the medial tissues.

After BAP and NPA application, no evident changes were detected in the DR5::GFP signal in the abaxial (external) side of the valveless gynoecia, compared to the wild type. The model proposed by Sessions in 1997 suggests that the apical-basal patterning of the gynoecia is determined through the specification of two boundaries that are specified very early, during floral stage 6 when the gynoecial primordium is a radially symmetric dome of cells (Sessions, 1997; Larsson et al., 2013). Based on this, one possible explanation is that changes in auxin signaling (*DR5::GFP)* may occur in early stages (stage 5–7) during BAP or NPA-treated gynoecium development causing the apical-basal defects and such changes cannot be detected at later stages of gynoecium development. In order to test this hypothesis it would be necessary to analyze auxin signaling during earlier valveless gynoecia development, which is technically challenging, or by using a more sensitive auxin signaling marker like the DII-VENUS sensor (Brunoud et al., 2012).

On the other hand, cytokinin negatively affects PIN expression and localization in the root meristem (Laplaze et al., 2007; Dello Ioio et al., 2008; Ruzicka et al., 2009). In contrast, here we observed that the auxin efflux carrier PIN1 expression was increased and localized in whole valveless gynoecia due to cytokinin application. This suggests that cytokinin has an opposite effect on PIN1 expression in the gynoecium versus the root meristem, as similarly observed in the root vasculature (Bishopp et al., 2011).

The cytokinin–auxin interaction can occur at different levels, i.e., cytokinin can affect auxin synthesis, transport or signaling, and auxin can affect cytokinin synthesis, degradation or signaling (Hwang et al., 2012; El-Showk et al., 2013). With the generated data so far we cannot rule out any of these possibilities related to apical-basal gynoecium patterning. However, because the NPA role is to block polar auxin transport and the phenotypes caused by both treatments were very similar, the observations obtained from our experiments suggest that the exogenous BAP application may be able to affect polar auxin transport and therefore cause apical-basal gynoecium patterning defects. Supporting this hypothesis is the observation that cytokinin can affect PIN expression and localization in gynoecia. Further support comes from the fact that the different auxin transport or signaling mutants tested in this work showed a similar sensitivity level for both treatments and the TCS::GFP and DR5::GFP expression pattern, respectively, were also similar for both treatments. Another possibility is that exogenous BAP application affects auxin on more than one action level and that the induced apical-basal gynoecium patterning defects are due to the sum of these changes. Future work should give more insights into the molecular mechanisms.

## AUTHOR CONTRIBUTIONS

Victor M. Zúñiga-Mayo and J. Irepan Reyes-Olalde performed experiments; all authors analyzed data; Victor M. Zúñiga-Mayo, Nayelli Marsch-Martinez, and Stefan de Folter drafted the manuscript. All authors provided intellectual content and contributed to manuscript revisions. All authors provided final approval of the manuscript. All authors agree to be accountable for all aspects of the work, including ensuring the accuracy and integrity of the work.

## Conflict of Interest Statement

The authors declare that the research was conducted in the absence of any commercial or financial relationships that could be construed as a potential conflict of interest.
